# Prognostic role of vitamin D receptor in breast cancer: a systematic review and meta-analysis

**DOI:** 10.1186/s12885-020-07559-w

**Published:** 2020-11-01

**Authors:** Haiyan Xu, Zhenhua Liu, Hongtai Shi, Chunbin Wang

**Affiliations:** 1 Department of Medical Oncology, The Second People’s Hospital of Lianyungang, 41 Hailian Road, Lianyungang, 222000 China; 2 Department of Radiotherapy, The First People’s Hospital of Yancheng, 66 Renmin Street, Yancheng, 224005 China; 3 Department of Radiotherapy, The Third People’s Hospital of Yancheng, 75 Juchang Street, Yancheng, 224005 China; 4 Department of Oncology, The Third People’s Hospital of Yancheng, 75 Juchang Street, Yancheng, 224005 China

**Keywords:** Breast cancer, Vitamin D receptor, Prognosis, Meta-analysis

## Abstract

**Background:**

A higher vitamin D intake improves the prognosis of early stage breast cancer (BC) patients. We hypothesized that vitamin D intake should refer to vitamin D receptor (VDR) expression. In order to prove this hypothesis, we first intend to evaluate the correlation between VDR expression and prognosis of BC patients using meta-analysis.

**Methods:**

Literatures from PubMed, Embase, and the Cochrane Library (last update by May 20, 2020) were retrieved to find studies assessing the prognostic role of VDR in BC. The hazard ratios (HRs) for patients’ survival were extracted for pooled analyses. Subgroup analysis, sensitivity analysis and meta-regression were performed to explore the sources of heterogeneity.

**Results:**

Seven articles containing eight studies with 2503 patients were enrolled. The results from the pooled analyses showed that the VDR expression generally had no relationship with BC patients’ overall survival (OS), disease-free survival (DFS), cancer-specific survival (CSS), and progression-free survival (PFS) (*P* > 0.05). Because only the number of studies exploring the relationship between VDR expression and OS is greater than five and there is heterogeneity, we explored the sources of heterogeneity of these studies. Subgroup analyses showed that the VDR expression in the nucleus had no relationship with OS, but high total VDR expression in the nucleus and cytoplasm was related to a better OS (pooled HR = 0.41; 95% CI = 0.18–0.95; *P* = 0.038). In addition, in subgroup of studies using cut-off values other than ‘immunoreactive score (IRS)>5’ and ‘IRS > 25′, high VDR expression was associated with a better OS (pooled HR = 0.47; 95% CI = 0.30–0.74; *P* = 0.001). Sensitivity analysis showed that the result pattern was not obviously affected by any single study. Meta-regression showed that the source of heterogeneity was not country (*P* = 0.657), pathological type (*P* = 0.614), molecular type (*P* = 0.423), staining location (*P* = 0.481), or cut-off value (*P* = 0.509).

**Conclusions:**

The protein expression level of VDR in entire BC cells evaluated by immunohistochemistry is related to the OS of BC patients. It is expected that a more individualized vitamin D intake and a more accurate prognosis assessment can be recommended for BC patients based on the VDR expression. Of course, more preclinical and clinical studies are needed.

## Background

The global incidence of breast cancer (BC) has been on the rise since the late 1970s, seriously threatening women’s health [1]. According to statistics [2], there were 2.088 million new cases of BC in the world in 2018, and the incidence of BC in developed countries was significantly higher than that in developing countries. BC has become one of the most common malignant tumors that causes female death [3]. In the past few decades, despite improvements in surgical techniques and changes in chemotherapy and radiotherapy methods, the mortality rate of BC has significantly decreased, but the prognosis of BC patients is still not satisfactory [4]. Previous meta-analyses have supported an inverse association between vitamin D status/intake and BC occurrence [5, 6], and an association of low levels of vitamin D with increased risk of recurrence and death in BC patients [7, 8].

Vitamin D is a steroid hormone in structure, and its metabolic active substance is 1,25 (OH)_2_D_3_, which plays an important role in calcium and phosphorus metabolism. Preclinical studies have found that 1,25 (OH)_2_D_3_ can inhibit the proliferation of BC cell lines and promote their differentiation and apoptosis [9, 10]. Moreover, findings from a prospective study including 10,578 premenopausal and 20,909 postmenopausal women suggested that higher intakes of calcium and vitamin D may reduce the risk of BC in premenopausal women [11]. Therefore, in our clinical work, we suggest that early BC patients and premenopausal women should appropriately increase their vitamin D intake. However, it seems inappropriate to recommend that each patient take the same dose of vitamin D, because vitamin D as a ligand for its biological function depends on binding to the receptor, while the vitamin D receptor (VDR) is expressed vary in different patients. Therefore, we established a scientific hypothesis that the vitamin D intakes of BC patients should refer to their VDR expression levels. To prove this hypothesis, we first need to verify the correlation between the VDR expression and the prognosis of BC patients.

VDR is a ligand-dependent transcriptional regulator protein and a member of the nuclear receptor superfamily [12, 13]. In the breast epithelium, vitamin D interacts with VDR in the same place or in adjacent cells to maintain differentiation and quiescence [14]. A case-control study by Hemida et al. showed that VDR expression was upregulated in BC tissues and correlated with estrogen receptor alpha (ER-α) expression [15]. Retrospective studies by Heublein [16] and Huss [17] et al. showed that low expression of VDR is an indicator for poor prognosis of BC. However, there are certain differences between the various studies, and the sample size of the studies is small, so the relevant results cannot directly and effectively guide clinical work. In view of this, a meta-analysis was carried out by collecting literatures on VDR expression in BC to clarify the relationship between VDR expression and the prognosis of BC patients.

## Methods

### Search strategy

The following databases were retrieved to find literatures: PubMed, Embase, and the Cochrane Library (last update by May 20, 2020). There were no language restrictions for literature collection. ‘Receptors, calcitriol’ and ‘breast neoplasms’ were the Medical Subject Heading (MeSH) terms. Each retrieved literature was read throughout.

#### Inclusion criteria

- Studies investigated the prognostic role of VDR in BC.
- Studies provided the hazard ratio (HR) and 95% confidence intervals (CI) of VDR or the survival curves.
- The patients did not receive neoadjuvant radiotherapy or chemotherapy before surgery.

#### Exclusion criteria

- Reviews, letters, case reports, animal trials and conference abstracts.
- Studies with small sample size (< 50).
- Only the complete or most recent study was enrolled if one patient cohort were researched by multiple studies.

### Data extraction

Some important parameters extracted from the included studies have been showed in the Table 1. If the article reports both univariate analysis results and multivariate analysis results, the latter will be adopted because it reduces the interference of confounding factors.

**Table 1:** Main characteristics of all studies included in the meta-analysis

Study	Country	Case number	High expression (%)	Pathological type	Molecular type	Staining location	Tumor stage	Follow-up (months)	Cut-off value	Multivariate analysis	HRs provided from	Outcome	NOS score
Heublein 2017 [16]	Germany	117	57 (48.7)	NR	Multiple	Nucleus	Multiple	Mean 81.8	IRS > 4.14	No	SC	OS	6
Huss 2019 [17]	Sweden	678	553 (81.6)	Multiple	Multiple	Nucleus	Multiple	NR	Positive cells > 10%	No	Report	CSS	8
Al-Azhri 2017 [18]	America	1114	283 (25.5)	NR	Multiple	Nucleus	Multiple	Median 72 (3–201)	IRS > 5	Yes	Report	OS/PFS/CSS	8
Ditsch 2012 [19]	Germany	82	75 (91.5)	Invasive ductal	Multiple	Nucleus and cytoplasm	NR	NR	IRS > 5	Yes	Report	OS/DFS	7
Soljic 2017 [20]	Yugoslavia	96	26 (27)	Multiple	Triple negative	Nucleus and cytoplasm	I-IIIa	Mean 69 (3–143)	NR	No	Report	OS/DFS	7
Ishida 2018 [21]	Japan	144	NR	NR	NR	Nucleus	I-III	Mean 7.7 (6–129)	NR	No	Report	DFS	8
Zehni (unifocal) 2019 [22]	Germany	155	66 (42.6)	Multiple	Multiple	Nucleus	NR	NR	IRS > 25	Yes	Report	OS/DFS	7
Zehni (multifocal) 2019 [22]	Germany	117	60 (51.3)	Multiple	Multiple	Nucleus	NR	NR	IRS > 25	Yes	Report	OS/DFS	7

*NR* Not report, *IRS* Immunoreactive score, *HR* Hazard ratio, *SC* Survival curve, *OS* Overall survival, *CSS* Cancer-specific survival, *PFS* Progression-free survival, *DFS* Disease-free survival, *NOS* Newcastle-Ottawa Quality Assessment Scale

### Guidelines and quality assessment

This meta-analysis complied with the Systematic Reviews and Meta-Analyses (PRISMA) guidelines [23]. The quality assessment of the studies followed the Newcastle-Ottawa Quality Assessment Scale (NOS). The lowest NOS score is 0 and the highest is 9. A score of 6 or more indicates high quality of the study.

### Statistical analysis

When the article did not directly report HR and 95% CI and only provided Kaplan-Meier survival curves, we indirectly extracted HR and 95% CI from the curves according to Tierney’ method [24]. The *P* value of chi-square test < 0.05 and/or *I*^2^ ≥ 25% indicated heterogeneity, then the random-effects model (the DerSimonian-Laird method) was used [25], otherwise the fixed-effects model (the Mante-Haenszel method) was used [26]. If there is heterogeneity, subgroup analysis, sensitivity analysis and meta-regression will be performed to explore the sources of heterogeneity. The STATA version 12.0 (Stata Corporation, College Station, TX, USA) was used to analysis data and generate figures. Except estimating heterogeneity and publication bias, a *P* value less than 0.05 was considered significant.

## Results

### Study characteristics

**Fig. 1:**
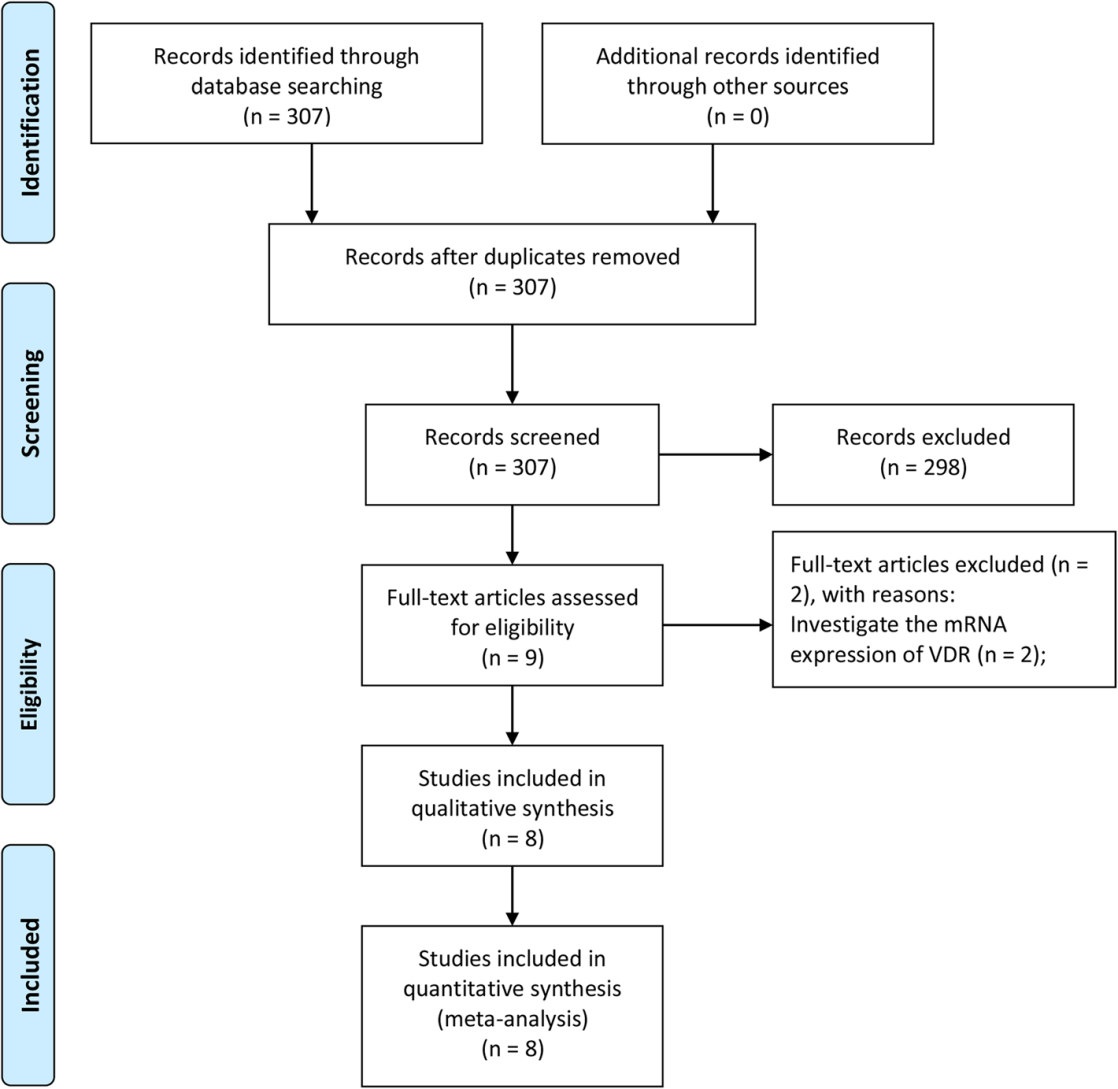
Flow diagram of the study selection process for the meta-analysis

Summarizing the search results of the three databases, 307 articles were initially screened out. Then 298 articles were excluded because their content did not include the prognostic value of VDR in BC. We read the full text of the remaining nine articles. We found that two remaining articles investigated the relationship between VDR mRNA expression and prognosis of BC patients. Since the data in these two articles came from public databases and was duplicated, they were excluded. Finally, seven articles containing eight studies with 2503 patients were included in this meta-analysis (Fig. 1) [16–22]. The article by Zehni et al. [22] contains two subgroup studies. The mean score of NOS for the eight studies was 7.25, ranging from 6 to 8 (Table 1). VDR expression was detected using immunohistochemistry (IHC) in all included studies.

Table 1 showed the important parameters of included studies. Patients came from five countries: Germany, Sweden, America, Yugoslavia or Japan. The number of patients in each study ranged from 82 to 1114. Patients in most included studies had multiple pathological types and molecular types. Four studies investigated the prognostic role of intranuclear VDR expression. Two studies investigated the prognostic role of total VDR expression in nucleus and cytoplasm. Four studies used immunoreactive score (IRS) to assess the protein expression of VDR. HRs for overall survival (OS) were extracted in six studies, five of which were reported directly. HRs for disease-free survival (DFS) were extracted in five studies, three of which were reported directly. HRs for cancer-specific survival (CSS) were reported directly in two studies. Only one study investigated patients’ progression-free survival (PFS).

### Overall survival

**Fig. 2:**
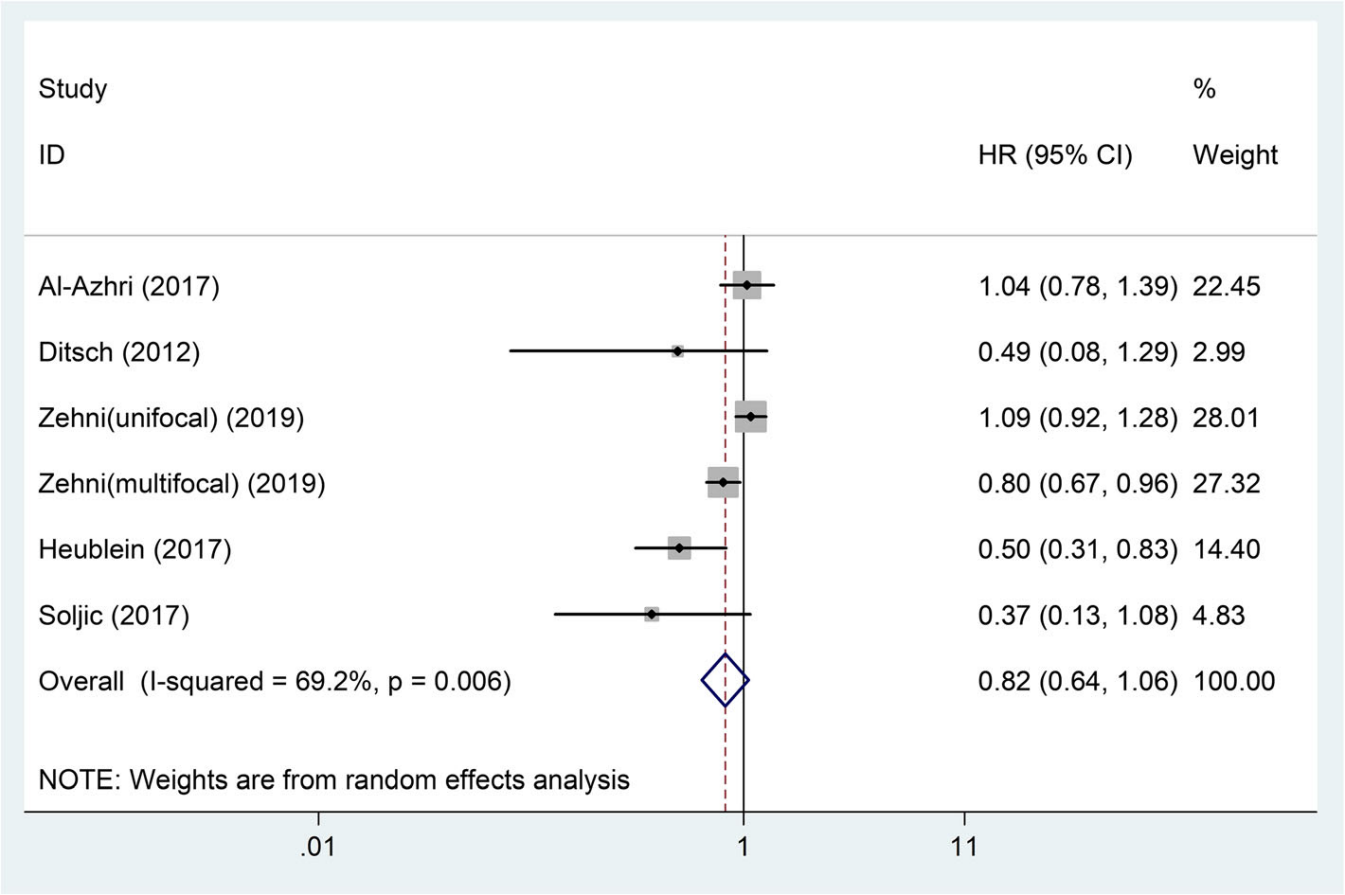
Forest plot of studies evaluating the hazard ratio of high VDR expression for the overall survival of breast cancer patients. VDR: vitamin D receptor; HR: hazard ratio; CI: confidence interval

Five articles containing six studies with 1681 patients investigated the relationship between VDR expression and patients’ OS in BC. Because the heterogeneity of these six studies existed (*I*^2^ = 69.2%, *P* = 0.006), a random-effects model was used. The results of pooled analyses were showed in Table 2. In general, the VDR expression had no relationship with BC patients’ OS (pooled HR = 0.82; 95% CI = 0.64–1.06; *P* = 0.052) (Fig. 2). Because the number of these studies is greater than five, we explored the sources of heterogeneity.

**Table 2:** The pooled associations between VDR expression and the prognosis of patients with breast cancer

Outcome subgroup	Study number	Case number	HR (95%CI)-model	*P* value	Heterogeneity	
					*I*^2^ (%)	*P*
OS	6	1681	0.82 (0.64–1.06)-random	0.052	69.2	0.006
Country						
Germany	4	471	0.80 (0.58–1.10)- random	0.161	763	0.005
Others	2	1210	0.71 (0.27–1.88)-random	0.488	70.7	0.065
Pathological type						
Invasive ductal	1	82	0.49 (0.12–1.97)	0.315	–	–
Others	5	1599	0.83 (0.64–1.08)- random	0.168	74.1	0.004
Molecular type						
Triple negative	1	96	0.37 (0.13–1.08)	0.066	–	–
Multiple	5	1585	0.85 (0.67–1.10)-random	0.229	70.1	0.010
Staining location						
Nucleus	4	1503	0.87 (0.68–1.12)-random	0.293	76.1	0.006
Nucleus and cytoplasm	2	178	0.41 (0.18–0.95)-fixed	0.038	0	0.753
Cut-off value						
IRS > 5	2	1196	0.98 (0.67–1.45)- fixed	0.931	7.3	0.299
IRS > 25	2	272	0.94 (0.70–1.26)-random	0.664	82.7	0.016
Others	2	213	0.47 (0.30–0.74)- fixed	0.001	0	0.613
DFS	5	594	1.11 (0.73–1.70)- random	0.625	72.7	0.005
CSS	2	1792	0.78 (0.42–1.46)- random	0.439	73.4	0.052
PFS	1	1114	1.14 (0.87–1.50)	0.346	–	–

*VDR* Vitamin D receptor, *OS* Overall survival, *IRS* Immunoreactive score, *DFS* Disease-free survival, *CSS* Cancer-specific survival, *PFS* Progression-free survival, *HR* Hazard ratio, *CI* Confidence interval

**Fig. 3:**
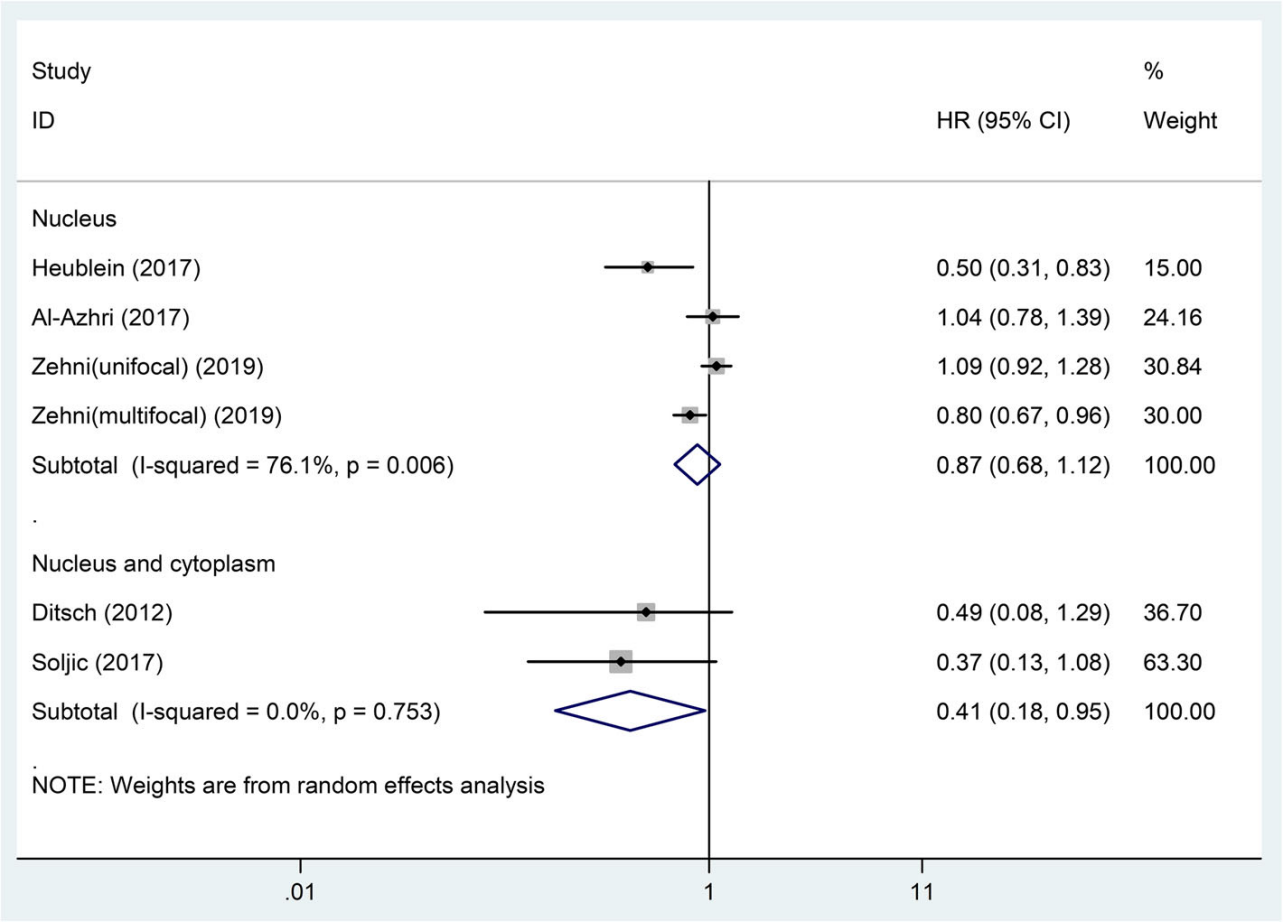
Forest plot of studies evaluating the hazard ratio of high VDR expression for the overall survival of breast cancer patients stratified by staining location. VDR: vitamin D receptor; HR: hazard ratio; CI: confidence interval

**Fig. 4:**
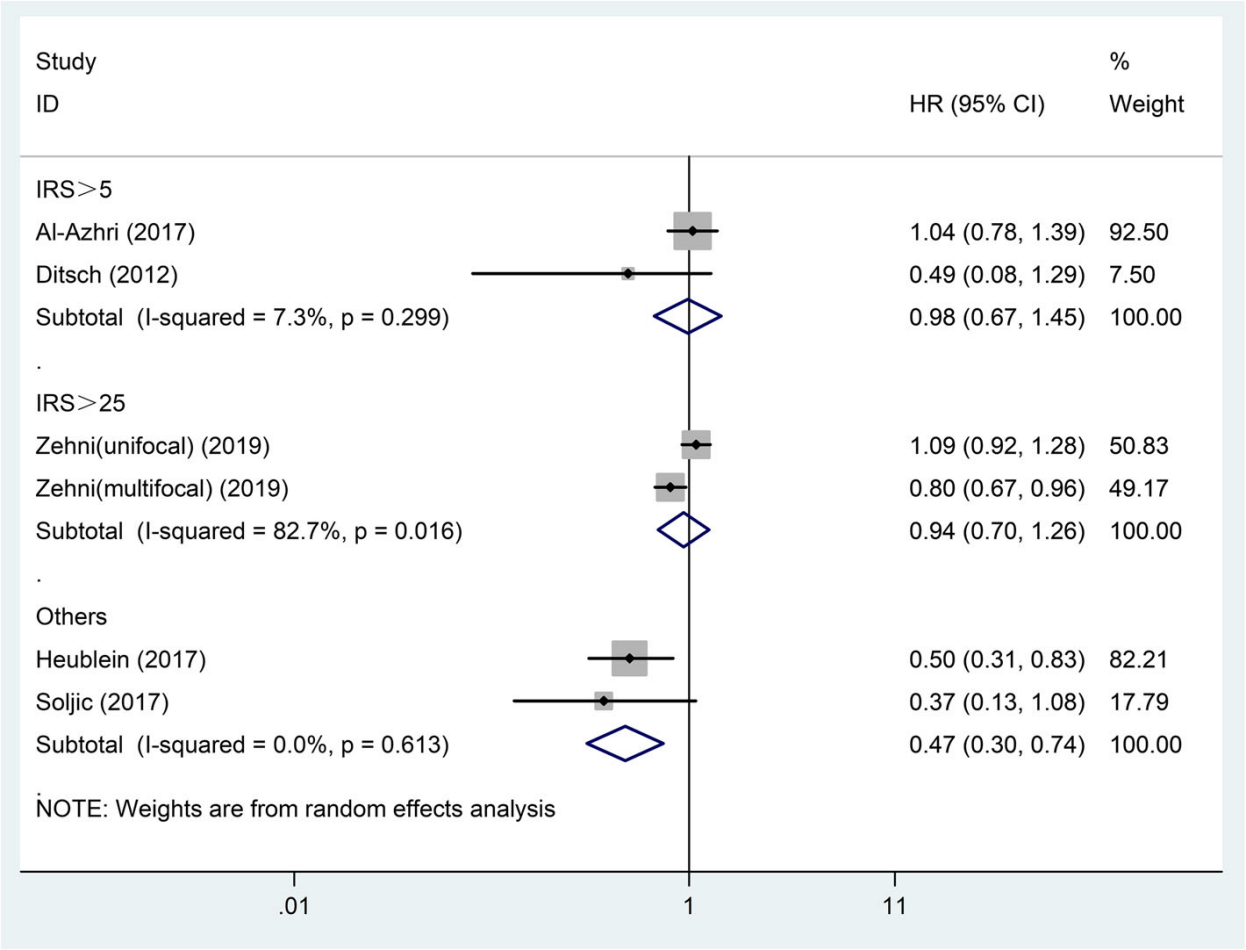
Forest plot of studies evaluating the hazard ratio of high VDR expression for the overall survival of breast cancer patients stratified by cut-off value. VDR: vitamin D receptor; IRS: immunoreactive score; HR: hazard ratio; CI: confidence interval

The results of subgroup analyses showed that in different subgroups divided by patients’ country, pathological type and molecular type, VDR expression remained unrelated to BC patients’ OS (*P* > 0.05, Table 2). However, in subgroups divided by staining location and cut-off value, positive results were observed. The VDR expression in the nucleus had no relationship with OS, but high total VDR expression in nucleus and cytoplasm was related to better OS (pooled HR = 0.41; 95% CI = 0.18–0.95; *P* = 0.038) (Table 2; Fig. 3). In subgroup of studies using cut-off values other than ‘IRS > 5′ and ‘IRS > 25′, high VDR expression was associated with better OS (pooled HR = 0.47; 95% CI = 0.30–0.74; *P* = 0.001) (Table 2; Fig. 4).

**Fig. 5:**
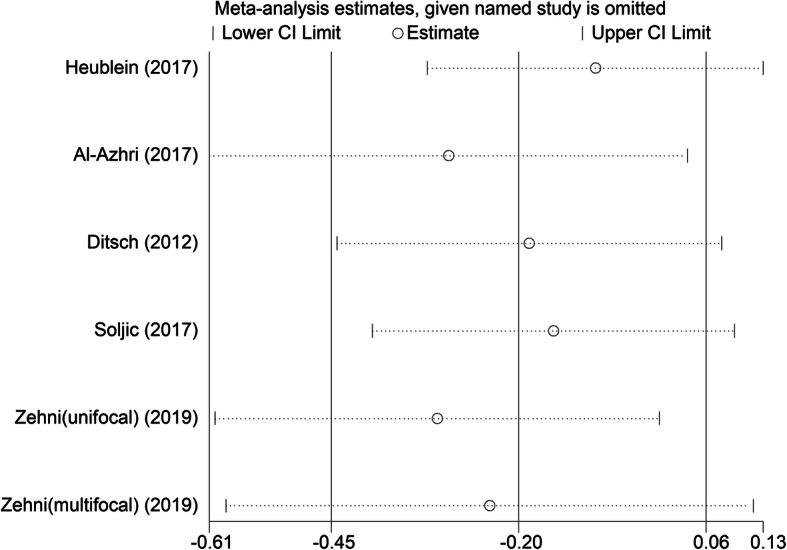
Sensitivity analysis of studies evaluating the relationship between VDR expression and patients’ overall survival in breast cancer. VDR: vitamin D receptor; CI: confidence interval

Sensitivity analysis using a random-effects model showed that the result pattern was not obviously affected by any single study (Fig. 5). Meta-regression showed that the source of heterogeneity was not country (*P* = 0.657), pathological type (*P* = 0.614), molecular type (*P* = 0.423), staining location (*P* = 0.481), or cut-off value (*P* = 0.509).

**Fig. 6:**
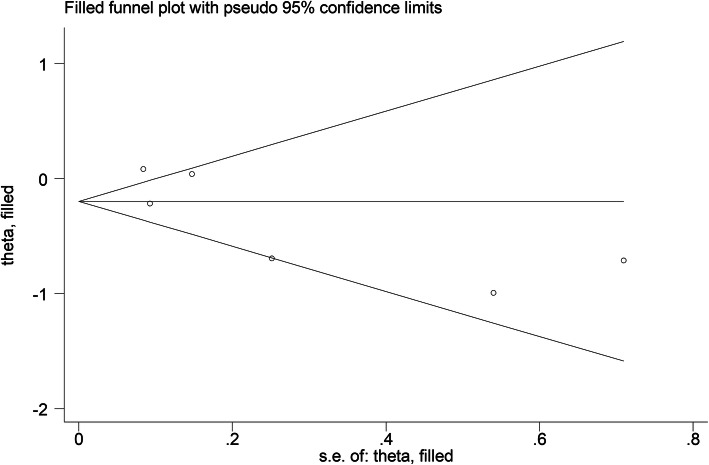
Funnel plot of publication bias for studies evaluating the relationship between VDR expression and patients’ overall survival in breast cancer

There was a publication bias because the funnel plot was asymmetrical (Fig. 6). The “Trim and Fill” method under a random-effects model was used to eliminate the publication bias [27]. After eliminating the publication bias, the VDR expression was still not related to BC patients’ OS in general (corrected pooled HR = 0.82; 95% CI = 0.64–1.06; *P* = 0.127).

### Disease-free survival

**Fig. 7:**
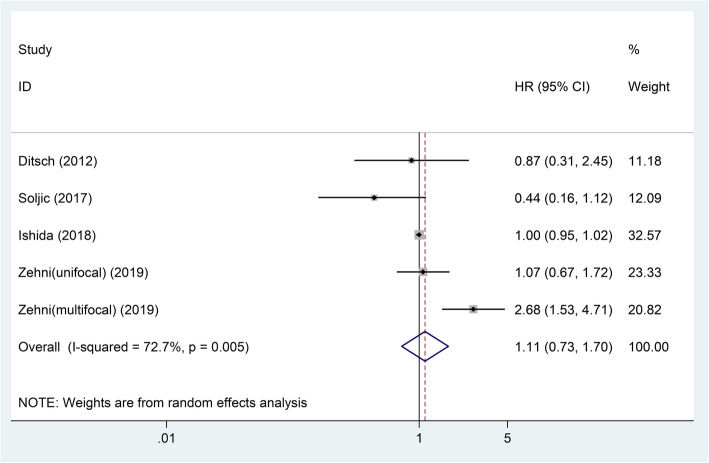
Forest plot of studies evaluating the hazard ratio of high VDR expression for the disease-specific survival of breast cancer patients. VDR: vitamin D receptor; HR: hazard ratio; CI: confidence interval

Four articles containing five studies with 594 patients investigated the relationship between VDR expression level and patients’ DFS in BC. Because the heterogeneity of these five studies existed (*I*^2^ = 72.7%, *P* = 0.005), a random-effects model was used. The pooled result showed that there was no relationship between VDR expression and patients’ DFS (pooled HR = 1.11; 95% CI = 0.73–1.70; *P* = 0.625) (Table 2; Fig. 7).

### Cancer-specific survival

Two studies with 1792 patients investigated the relationship between VDR expression level and patients’ CSS in BC. Because the heterogeneity of these two studies existed (*I*^2^ = 73.4%, *P* = 0.052), a random-effects model was used. The pooled result showed that there was no relationship between VDR expression and patients’ CSS (pooled HR = 0.78; 95% CI = 0.42–1.46; *P* = 0.439) (Table 2).

### Progression-free survival

Only one study containing 1114 patients investigated the relationship between VDR expression and patients’ PFS in BC. There was no relationship between VDR expression and patients’ PFS (HR = 1.14; 95% CI = 0.87–1.50) (Table 2).

## Discussion

It has been reported that vitamin D can be regarded as a protective factor for reducing the risk of various cancers including BC, and can inhibit the cell proliferation of normal and malignant breast cells [28], and induce cell differentiation and apoptosis [29]. Vitamin D is involved in the process of regulating cell growth, differentiation, and apoptosis by binding to VDR [30]. VDR is a nuclear receptor that regulates gene expression and is expressed in 80 to 90% of BC patients [31]. VDR can be expressed in breast epithelial cells, which suggests that vitamin D may directly affect the sensitivity of the glands. In vitro studies have shown that the VDR ligand, 1,25 (OH)_2_D_3_, is involved in maintaining the differentiation of breast cells. Knocking out the VDR gene increases the susceptibility of BC in mice. The expression of VDR is down-regulated in invasive BC [30], suggesting that the expression of VDR is negatively correlated with the progress of BC, and the expression of VDR has a certain protective effect on the breast. Therefore, in theory, the high expression of VDR in BC should be related to a good prognosis.

In this meta-analysis, we found that the relationship between VDR expression and prognosis in BC was mainly affected by the staining location. Results of the subgroup analysis showed that only the total VDR expression in nucleus and cytoplasm was related to BC patients’ survival. VDR mainly functions as a nuclear receptor [32, 33], but it is widely distributed on multiple subcellular structures, including the nucleus, nuclear membrane, cytoplasm, and cell membrane [17]. Our results indicate that VDR in the cytoplasm also exerts specific biological functions in the progression of BC cells. In view of this, we recommend that when performing immunohistochemical analysis of BC specimens in clinical work, the total expression of VDR in nucleus and cytoplasm should be detected instead of only the expression of VDR in nucleus.

Our study has made it clear that both the serum vitamin D level and the expression of VDR are related to the prognosis of BC patients, which suggests that the serum vitamin D level of different BC patients should be adjusted according to the expression of VDR. BC patients with high total VDR expression in nucleus and cytoplasm may not need too much vitamin D intake. Of course, this hypothesis needs to be further verified by controlled clinical trials with larger sample sizes. In addition, our results are also conducive to more accurate assessment of the prognosis of BC patients, which is important for formulating appropriate treatment plans.

At present, Tumor Node Metastasis (TNM) staging is the most important indicator for assessing the prognosis of BC patients, but the accuracy of prediction is reduced due to individual differences. It is well known that patients with same TNM staging may have different prognosis. Therefore, an effective biological indicator is urgently needed to help assess the prognosis of BC patients [20]. Although serum vitamin D level and VDR expression are both related to the prognosis of BC patients [7, 11], VDR expression seems a more suitable prognostic indicator of BC because serum vitamin D level fluctuates greatly due to diet and sunlight exposure.

In addition to the prognostic value of VDR protein expression, the prognostic value of VDR mRNA expression in BC has also been reported [34, 35]. Murray et al. [34] evaluated a pooled database of 12 publicly available BC datasets (*n* = 2592 patients) containing gene expression data, then found that the mRNA expression of VDR was not related to the DFS of BC patients as a whole. In this meta-analysis, we found that the protein expression of VDR was also not related to the DFS of BC patients. This suggests that the mRNA expression and the protein expression of VDR may be consistent. However, there is still a lack of reports on the relationship between VDR mRNA expression and OS of BC patients. The correlation between VDR polymorphism and BC has also been researched in previous studies [36–38]. Raimondi et al. reported that the polymorphism of the third gene Bsml and fifth gene Fokl of the VDR gene may be able to regulate the risk of BC [39]. A high-quality meta-analysis showed that the Fokl polymorphism of the VDR gene was associated with an increased risk of BC [37]. However, another meta-analysis by Lu et al. showed that VDR polymorphism (Fok1, Bsm1, Taq1, and Apa1) were not associated with the risk of BC in general population [36]. The roles of VDR mRNA expression and polymorphism in BC need to be further explored by more prospective clinical studies.

Our meta-analysis is the first to study the relationship between VDR protein expression level and BC prognosis. Although only 8 studies were included, the present meta-analysis based on the data of 2503 patients can still provide some help and reference for assessing the prognostic role of VDR expression in BC. Of course, the small number of included studies may affect the reliability of the results of the subgroup analysis. In addition, this meta-analysis also has some other shortcomings. For example, part of the HRs is obtained from univariate analyses, which will overestimate the effect size because the influence of confounding factors is not excluded. The clinical information provided by the included studies is inadequate. Some studies did not provide the pathological and molecular types of patients, which prevented us from performing high-quality subgroup analyses based on these clinical features, leading to the omission of some valuable positive results. Furthermore, some HRs were estimated based on survival curves, which caused statistical errors.

## Conclusion

Previous meta-analyses have shown an inverse association between serum vitamin D level and BC prognosis. Similarly, VDR expression is also related to the prognosis of BC patients. Our review and meta-analysis demonstrated that BC patients with high total VDR expression in nucleus and cytoplasm had better OS. This is an important finding in terms of future randomized controlled trials which might reveal a directive function of VDR expression for the adjustment of vitamin D intake in BC patients. VDR expression is not affected by diet and sunlight exposure, so it is more suitable for assessing the prognosis of BC than the serum vitamin D level. A more accurate assessment of the prognosis by combining TNM staging and VDR expression is conducive to formulating a more appropriate treatment plan for BC patients. VDR expression is expected to become a routine immunohistochemical examination item in the pathological diagnosis of BC.

## Data Availability

Meta-analysis is a secondary analysis, which the data are all fully available without restriction, and all the material can be found in the included original studies.

## Abbreviations

BC: Breast cancer
VDR: Vitamin D receptor
MeSH: Medical Subject Heading
IHC: Immunohistochemistry
HR: Hazard ratio
CI: Confidence intervals
TNM: Tumor Node Metastasis
NOS: Newcastle-Ottawa Quality Assessment Scale
IRS: Immunoreactive score
OS: Overall survival
DFS: Disease-free survival
CSS: Cancer-specific survival
PFS: Progression-free survival
